# Effects of peak ankle dorsiflexion angle on lower extremity biomechanics and pelvic motion during walking and jogging

**DOI:** 10.3389/fneur.2023.1269061

**Published:** 2024-02-01

**Authors:** Yi Rao, Nan Yang, Tianyu Gao, Si Zhang, Haitao Shi, Yiqun Lu, Shuang Ren, Hongshi Huang

**Affiliations:** ^1^Department of Rehabilitation, Xiyuan Hospital, China Academy of Chinese Medical Sciences, Beijing, China; ^2^Department of Sports Medicine, Peking University Third Hospital, Institute of Sports Medicine of Peking University, Beijing Key Laboratory of Sports Injuries, Engineering Research Center of Sports Trauma Treatment Technology and Devices, Ministry of Education, Beijing, China; ^3^College of Exercise and Health Sciences, Tianjin University of Sport, Tianjin, China

**Keywords:** ankle dorsiflexion, kinematics, kinetic, gait, jogging

## Abstract

**Objective:**

Ankle dorsiflexion during walking causes the tibia to roll forward relative to the foot to achieve body forward. Individuals with ankle dorsiflexion restriction may present altered movement patterns and cause a series of dysfunction. Therefore, the aim of this research was to clearly determine the effects of peak ankle dorsiflexion angle on lower extremity biomechanics and pelvic motion during walking and jogging.

**Method:**

This study involved 51 subjects tested for both walking and jogging. The motion capture system and force measuring platforms were used to synchronously collect kinematics and kinetics parameters during these activities. Based on the peak ankle dorsiflexion angle during walking, the 51 subjects were divided into a restricted group (RADF group, angle <10°) and an ankle dorsiflexion-unrestricted group (un-RADF group, angle >10°). Independent-Sample *T*-tests were performed to compare the pelvic and lower limb biomechanics parameters between the groups during walking and jogging test on this cross-sectional study.

**Results:**

The parameters that were significantly smaller in the RADF group than in the un-RADF group at the moment of peak ankle dorsiflexion in the walking test were: ankle plantar flexion moment (*p* < 0.05), hip extension angle (*p* < 0.05), internal ground reaction force (*p* < 0.05), anterior ground reaction force (*p* < 0.01), pelvic ipsilateral tilt angle (*p* < 0.05). In contrast, the external knee rotation angle was significantly greater in the RADF group than in the un-RADF group (*p* < 0.05). The parameters that were significantly smaller in the RADF group than in the un-RADF group at the moment of peak ankle dorsiflexion in the jogging test were: peak ankle dorsiflexion angle (*p* < 0.01); the anterior ground reaction force (*p* < 0.01), the angle of pelvic ipsilateral rotation (*p* < 0.05).

**Conclusion:**

This study shows that individuals with limited ankle dorsiflexion experience varying degrees of altered kinematics and dynamics in the pelvis, hip, knee, and foot during walking and jogging. Limited ankle dorsiflexion alters the movement pattern of the lower extremity during walking and jogging, diminishing the body’s ability to propel forward, which may lead to higher injury risks.

## 1. Introduction

The range of motion of ankle dorsiflexion was defined as the talus rolls forward relative to the leg and at the same time slides posteriorly (talocrural dorsiflexion) (1). Adequate ankle dorsiflexion range of motion is necessary for daily functional activities such as walking, jogging, landing, and walking up and down stairs (2). During the stance phase of gait, dorsiflexion reaches the peak just before heel rise. It was shown that the magnitude of ankle dorsiflexion varies among individuals, it is generally in the range of 5–15 degrees, with a minimum of 10 degrees reported by Root et al. (3). Ankle dorsiflexion during walking causes the tibia to roll forward relative to the foot to achieve body forward (1, 4). Jogging, on the other hand, requires a greater angle of ankle dorsiflexion to achieve forward rolling (5).

Reduced ankle dorsiflexion is primarily caused by tightness in the gastrocnemius and soleus and insufficient posterior gliding of the talus and is also associated with musculoskeletal injuries of the foot and ankle joint (6). Some researchers have identified ankle dorsiflexion restriction has been indicated as a dangerous factor for lower extremity injuries (7–10) and can lead to compensatory movements that alter lower extremity movement patterns and generate excessive stress. These biomechanical changes can result in injuries such as plantar fasciitis (8, 11), Achilles tendinitis (12), and knee injuries due to altered knee alignment (13, 14). Moreover, limited ankle dorsiflexion leads to changes in pelvic movement patterns (15), and studies have indicated (16) that the lumbar-pelvic movement patterns are altered in patients with low back pain compared to those without low back pain, and that inadequate individual control of gait and abnormal lower limb biomechanics can produce excessive stress on the upper lumbosacral region, leading to the development of low back pain (17–19). Previous studies have shown that a decrease in ankle dorsiflexion angle leads to an increase in foot progression angle during the gait cycle (20), an earlier heel-off time (21), and a shorter stride length (22). From the point of view of the coupling pattern of the kinematic chain, the movement of the ankle may affect the temporal and movement parameters of the knee, hip, and pelvis (23). Inadequate ankle dorsiflexion affects the ability to move forward (24), preferring to land on the arch of the foot and the front foot, affecting ground reaction forces and the torque of the lower extremity joints (25, 26). It also alters peak hip and knee flexion and pelvic movement patterns during the swing phase (15). Therefore, the limitation of ankle dorsiflexion restriction can cause a series of dysfunction, and it is important to clarify the specific effect of ankle dorsiflexion angle on lower extremity biomechanics and pelvic movement for the prevention and treatment of functional impairment.

The influence of limited ankle dorsiflexion on certain lower extremity joint motion biomechanical parameters during walking has been investigated in the literature, but no study has yet investigated the effect of different angular range on overall lower extremity biomechanics as well as pelvic motion during jogging and further compared it with lower extremity biomechanics during walking. Therefore, the aim of this research was to compare the lower limb and pelvic biomechanics during the stance phase of gait between individuals with lower and higher peak ankle dorsiflexion angle and clearly determine the effects of different peak ankle dorsiflexion angle on the kinematics and kinetics of the hip, knee, and ankle joints in different planes of motion during walking and jogging, as well as on pelvic motion. The main hypothesis of this study is that individuals with limited peak ankle dorsiflexion angles have reduced knee and hip motion in the sagittal plane during walking and jogging tests, altered pelvic motion patterns, and reduced ground reaction forces corresponding to peak moments of ankle dorsiflexion in gait.

## 2. Materials and methods

### 2.1. Participants

This study was approved by the Ethics Committee of Peking University Third Hospital. And the study authorization number was M2023360 (June 23, 2023). All participants read and signed an approved informed consent document before data collection. 51 subjects (35 men and 16 women) volunteered for this cross-sectional study. The inclusion criteria were (1) age 18–40, BMI (body mass index) in the normal range (18.5–24.9) (2) no neurological disorders (3) no musculoskeletal disorders within the last 6 months that limited their physical activity (4) no surgery or acute injury history to the lower extremities or pelvis. All of these 51 subjects had sufficient physical strength to perform at least 5 sessions of walking and jogging tests and no complaints of pain or discomfort during data collection. 13 subjects showed limited dorsiflexion in squatting, which is defined as that the knee joint could not fully flexed or the heel would have to raise during squatting with the feet shoulder-width apart. The other 38 people were able to complete the squat test successfully without limited dorsiflexion.

Previous research experiments have used ankle dorsiflexion range of motion measurement techniques mostly in passive flexion of the ankle joint under non-weight-bearing conditions (3, 15, 20) or using a weight-bearing lunge position for measurement (27, 28). In contrast, the present study innovatively selected the peak ankle dorsiflexion angle during the support phase of the walking test as the criterion for differentiating whether subjects had limited ankle dorsiflexion. This method can more accurately confirm whether an individual has an appropriate ankle range of motion during walking or other functional movements.

Fifty-one subjects were divided into groups based on the peak ankle dorsiflexion angle during the support phase of the walking test. Subjects with a peak ankle dorsiflexion angle of less than 10° on either side during the walking test were included in the ankle dorsiflexion-restricted group (ankle dorsiflexion angle less than 10°, *n* = 30, hereinafter referred to as RADF group), while other subjects were included in the ankle dorsiflexion-unrestricted group (ankle dorsiflexion angle greater than 10°, *n* = 21, hereinafter referred to as un-RADF group). The sample size was calculated using G*Power software in this study, with an α level of 0.05 and statistical power of 80%, and an estimated effect size of 1.0. Based on the difference between groups on the main outcome measures peak knee external rotation obtained in a pilot study with ten individuals. A minimum of 20 participants per group was needed to detect between-subject differences. 10 subjects in the pilot study were from the Outpatient Department of Sports Medicine, Peking University Third Hospital. They all received ankle physical examination and questionnaire survey, and 5 subjects were limited in squatting.

### 2.2. Data collection

The subject’s static and dynamic 3D motion information was collected with an 8-camera infrared high-speed motion capture system (Vicon, T40) at 100 Hz. Kinetic parameters were collected with 2 3D force platforms (AMTI, BP400600) at 1000 Hz. Kinematic and kinetic data were synchronized by a synchronization box (AMTI, GEN5). Subjects were labeled with reflective marker dots on the bony parts and the model was optimized using the international general model plug-in-gait.

Subjects wore exercise shorts to fully expose the waist and mid-thigh below. After the reflective markers were fixed, subjects followed the test procedure to first familiarize themselves with the collection exercise requirements and process. The subjects stood in the center of the chamber with their feet shoulder-width apart and both upper extremities placed naturally on both sides of the body, maintaining a neutral position of the talofibular joint for three static tests to collect static data for defining the coordinate system of the skeletal segments. Subsequently, the subjects were tested by walking and jogging at a self-selected speed. The interval between the two tests was such that the subjects did not feel exerted. 5 valid data were collected for each movement and the average of the 5 tests was used for analysis. The whole tests were carried out in a space of 10 m long, 8 m wide and 3 m high, and the length of test tracking area was about 6 m.

### 2.3. Data processing

The lower extremity kinematic data from the subjects’ walking test and jogging test were processed, and the subjects were divided into the RADF group (<10°, *n* = 30; 22 men) and un-RADF group (>10°, *n* = 21; 8 men) according to the peak ankle dorsiflexion angle during the support period in the walking test. The biomechanical model of the rigid body was developed using a static test with the talocrural joint in a neutral position. The force platform determines the occurrence of heel-strike and toe-off the ground by using ground reaction forces to determine the stance phase of the entire gait process. The coordinate data were filtered using a low-pass butter-worth filter at 12 Hz. The ground-reaction force data were filtered using a lowpass butter-worth filter at 100 Hz. Time-series data for the kinematics and kinetics variables in the coronal, sagittal, and horizontal planes of the pelvis, hip, knee, and ankle joints were calculated using Visual 3D software (Cmotion, Germantown, MD version v6.00.18).

### 2.4. Data analysis

All statistical analyses were completed using SPSS 26.0 (IBM, New York, USA). Quantitative data were first tested for normality, and if they conformed to a normal distribution, they were expressed as mean ± standard deviation and subjected to a two-sample *t*-test; if they did not conform to a normal distribution, they were expressed as median and quartiles and subjected to a two-sample rank sum test. The significance level was set at a class I error probability of no greater than 0.05.

## 3. Results

### 3.1. Participant information

A total of 51 subjects participated in the study, including 25 men and 16 women. The RADF group (<10°, *n* = 30; 22 men) and un-RADF group (>10°, *n* = 21; 8 men). 17 of the 38 subjects who were not limited in squatting were classified in the RADF group based on the results of the walking test. The 30 subjects in the RADF group included 13 with passive limited dorsiflexion and 17 without passive limited dorsiflexion during squat test. There was no significant difference in age, height, and weight between the RADF group and un-RADF group (*p* > 0.05; see Table 1).

**Table 1:** Participant information.

Variables	un-RADF group (SD)	RADF group (SD)	*t*-value	*p*-value	Mean difference (95% CI)
Height (cm)	170.95 (5.84)	173.07 (8.43)	−0.99	0.33	2.11 (−2.17 to 6.39)
Body mass (kg)	70.62 (11.81)	72.48 (13.43)	−0.51	0.61	1.86 (−5.46 to 9.17)
Age (years)	26.38 (8.70)	29.83 (10.18)	−1.26	0.21	3.45 (−2.04 to 8.94)

SD, standard deviation; CI, Confidence Interval.

### 3.2. Walking

**Figure 1:**
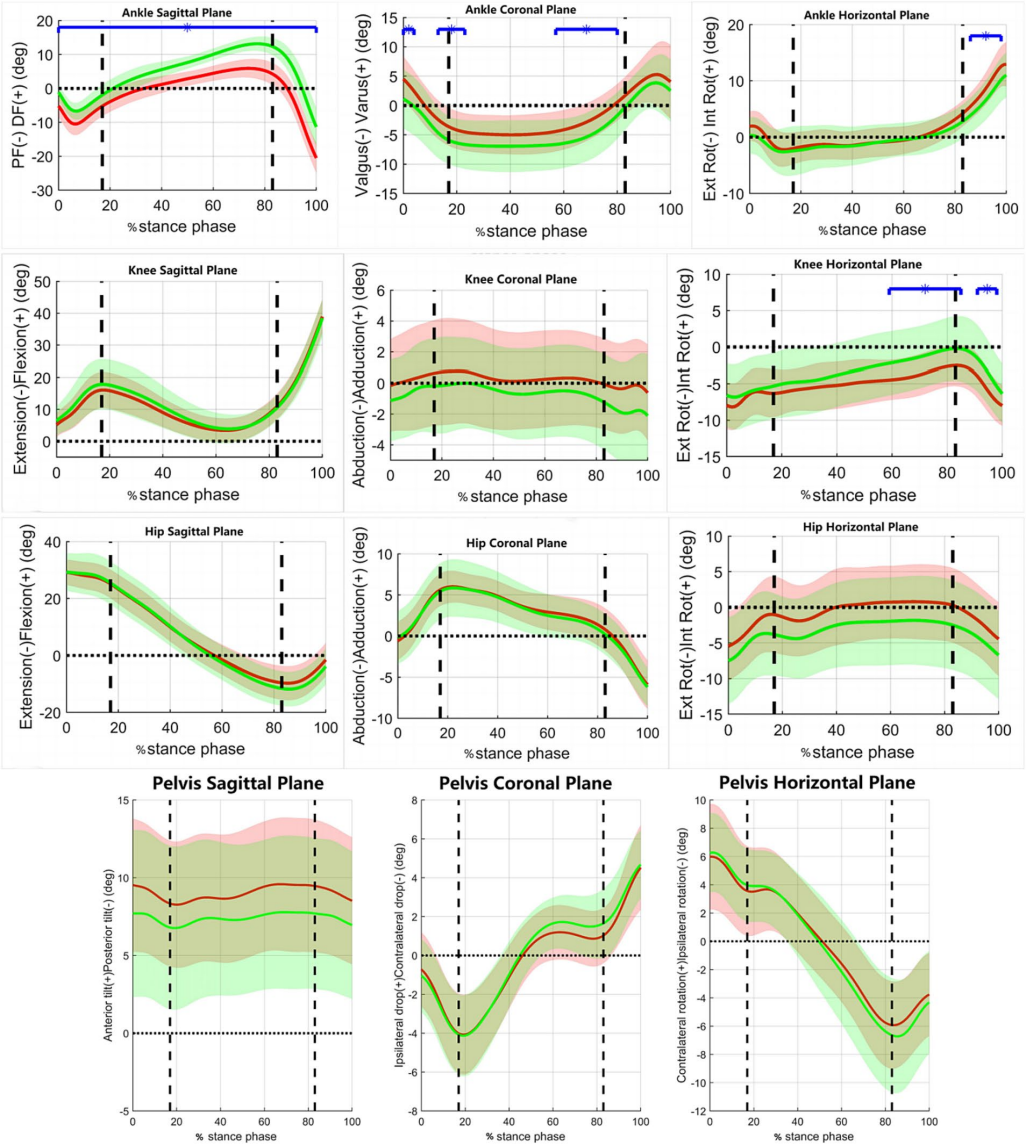
The variations of joint motion angles during walking in the two groups of subjects. *x*-axis, the percentage of the stance phase of gait; *y*-axis, Joint angles (°); Red Line, RADF group; Green Line, un-RADF group; Blue horizontal line, significant effect; DF-dorsiflexion, PF-plantarflexion; Ext Rot, External Rotation; Int Rot, Internal Rotation. First vertical dotted line, contralateral toe off; Second vertical dotted line, contralateral heel off.

**Figure 2:**
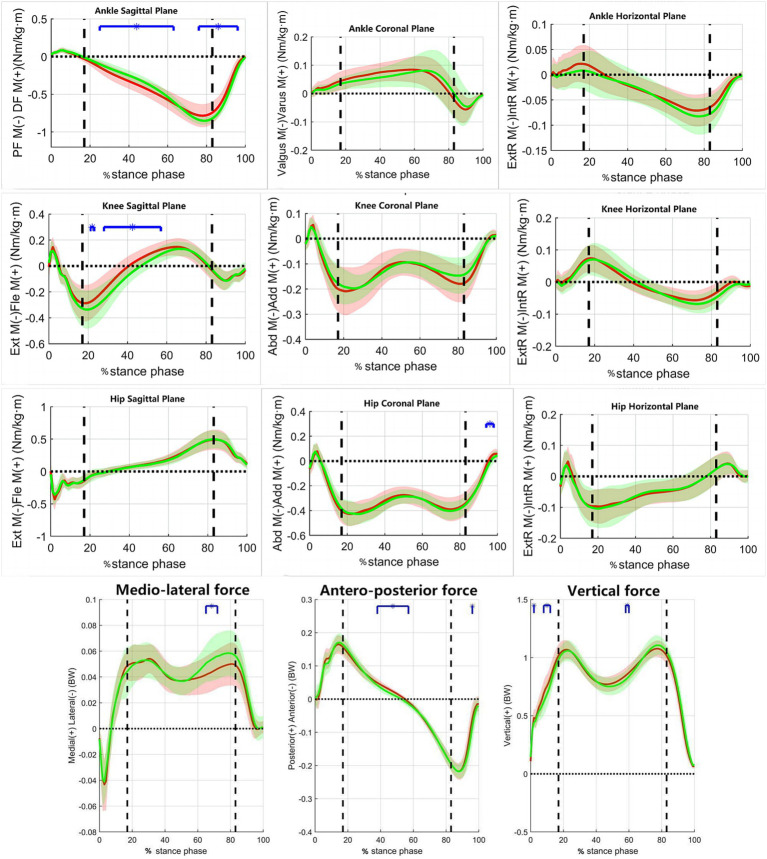
The variations of moments and ground-reaction force during walking in the two groups of subjects. *x*-axis, the percentage of the stance phase of gait; *y*-axis, the moment of force/Ground reaction force; Red Line, RADF group; Green Line, un-RADF group; Blue horizontal line, significant effect; BW, body weight; BW * BH, body weight multiplied by body height; Ext M, Extension moment; Fle M, Flexion moment; ExtR M, External Rotation moment; IntR M, Internal Rotation moment. First vertical dotted line, contralateral toe off; Second vertical dotted line, contralateral heel off.

Figure 1 shows the variations of joint motion angles in the coronal, sagittal, and horizontal planes of the pelvis and lower extremities during the stance phase of the walking process in the two groups of subjects. Figure 2 shows the variations of moments in the coronal, sagittal, and horizontal planes of each joint of the lower extremity during walking in the two groups of subjects. Table 2 shows the results of comparing the lower limb biomechanical parameters at the moment of peak ankle dorsiflexion angle during the stance phase of gait. The parameters that were significantly smaller in the RADF group than in the un-RADF group were: peak ankle dorsiflexion angle (RADF group: 6.20 ± 2.59°, un-RADF group: 13.52 ± 1.96°, *p* < 0.01); ankle plantarflexion moment corresponding to this peak moment (RADF group: 0.75 ± 0.15 BW* BH, un-RADF group: 0.84 ± 0.05 BW* BH, *p* < 0.05), hip extension angle (RADF group: 5.73 ± 6.72°, un-RADF group: 9.93 ± 6.21°, *p* < 0.05), internal ground reaction force (RADF group: 0.05 ± 0.02 BW, un-RADF group: 0.06 ± 0.02 BW, *p* < 0.05), anterior ground reaction force (RADF group: 0.10 ± 0.04 BW, un-RADF group: 0.14 ± 0.02 BW, *p* < 0.01), and pelvic ipsilateral tilt angle (RADF group: 0.82 ± 1.53°, un-RADF group: 1.81 ± 1.66°, *p* < 0.05). In contrast, the external knee rotation angle was significantly greater in the RADF group than in the un-RADF group (RADF group: 3.34 ± 2.84°, un-RADF group: 1.04 ± 4.46°, *p* < 0.05). No significant differences were found between other biomechanical parameters of the lower extremities and the pelvis.

**Table 2:** Biomechanical parameters during the stance phase during walking.

Variables	un-RADF group (SD)	RADF group (SD)	*t*-value	*p*-value	Mean difference (95% CI)
Ankle					
Ankle dorsiflexion (°)	13.52 (1.96)	6.2 (2.59)	10.95	<0.001*	−7.33 (−8.67 to −5.98)
Ankle eversion (°)	2.79 (3.75)	1.88 (3.64)	0.87	0.39	0.91 (−1.20 to 3.02)
Ankle adduction (°)	0.88 (3.05)	1.08 (2.79)	−0.25	0.81	0.2 (−1.45 to 1.86)
Ankle moment					
Ankle plantarflexion moment (BW * BH)	0.84 (0.05)	0.75 (0.15)	2.53	0.02*	0.09 (0.02 to 0.16)
Ankle varus moment (BW * BH)	0.05 (0.08)	0.06 (0.05)	−0.23	0.82	0.004 (−0.03 to 0.04)
Ankle abduction moment (BW * BH)	0.08 (0.04)	0.07 (0.03)	1.23	0.22	0.01 (−0.01 to 0.03)
Knee					
Knee flexion (°)	6.84 (4.48)	6.21 (4.09)	0.53	0.6	−0.64 (−3.07 to 1.80)
Knee abduction (°)	0.84 (3.12)	−0.24 (3.08)	1.23	0.23	1.08 (−0.69 to 2.85)
Knee external rotation (°)	1.04 (4.46)	3.34 (2.84)	−2.25	0.03*	−2.30 (−4.36 to −0.25)
Knee moment					
Knee flexion moment (BW * BH)	0.06 (0.08)	0.09 (0.08)	−1.05	0.3	0.02 (−0.02 to 0.07)
Knee abduction moment (BW * BH)	0.13 (0.07)	0.15 (0.07)	−0.81	0.42	−0.02 (−0.06 to 0.02)
Knee external rotation moment (BW * BH)	0.06 (0.03)	0.05 (0.03)	1.15	0.26	0.01 (−0.01 to 0.03)
Hip					
Hip extension (°)	9.93 (6.21)	5.73 (6.72)	2.27	0.03*	4.20 (0.47 to 7.92)
Hip adduction (°)	1.13 (2.59)	2.4 (2.38)	−1.81	0.08	1.27 (−0.14 to 2.68)
Hip external rotation (°)	2.3 (6.50)	−0.46 (5.22)	1.68	0.10	2.76 (−0.54 to 6.06)
Hip moment					
Hip flexion moment (BW * BH)	0.44 (0.13)	0.37 (0.15)	1.68	0.10	−0.07 (−0.15 to 0.01)
Hip abduction moment (BW * BH)	0.39 (0.08)	0.38 (0.09)	0.56	0.58	0.01 (−0.04 to 0.06)
Hip external rotation moment (BW * BH)	0.002 (0.05)	0.02 (0.05)	−1.53	0.13	−0.02 (−0.05 to 0.01)
Pelvis					
Anterior pelvic tilt (°)	7.79 (5.07)	9.53 (4.36)	−1.32	0.19	1.74 (−0.92 to 4.41)
Pelvic ipsilateral tilt (°)	1.81 (1.66)	0.82 (1.53)	2.19	0.03*	−0.99 (−1.89 to −0.08)
Pelvis ipsilateral rotation (°)	5.96 (4.32)	4.08 (3.27)	1.77	0.08	1.88 (−0.26 to 4.01)
GRF					
Medial (BW)	0.06 (0.02)	0.05 (0.02)	2.13	0.04*	−0.01 (−0.02 to −0.001)
Anterior (BW)	0.14 (0.02)	0.1 (0.04)	3.67	0.001*	0.04 (0.02 to 0.06)
Vertical (BW)	1.09 (0.09)	1.05 (0.08)	1.83	0.07	−0.04 (−0.09 to 0.004)
Walking velocity					
V (m/s)	1.26 (0.11)	1.28 (0.10)	−0.47	0.64	0.01 (−0.05 to 0.08)

SD, standard deviation; CI, Confidence Interval; *, significant effect; BW, body weight; BW * BH, body weight multiplied by body height; GRF, ground-reaction force; Medial, medial ground reaction force; Anterior, anterior ground reaction force; Vertical, Vertical ground reaction force.

### 3.3. Jogging

**Figure 3:**
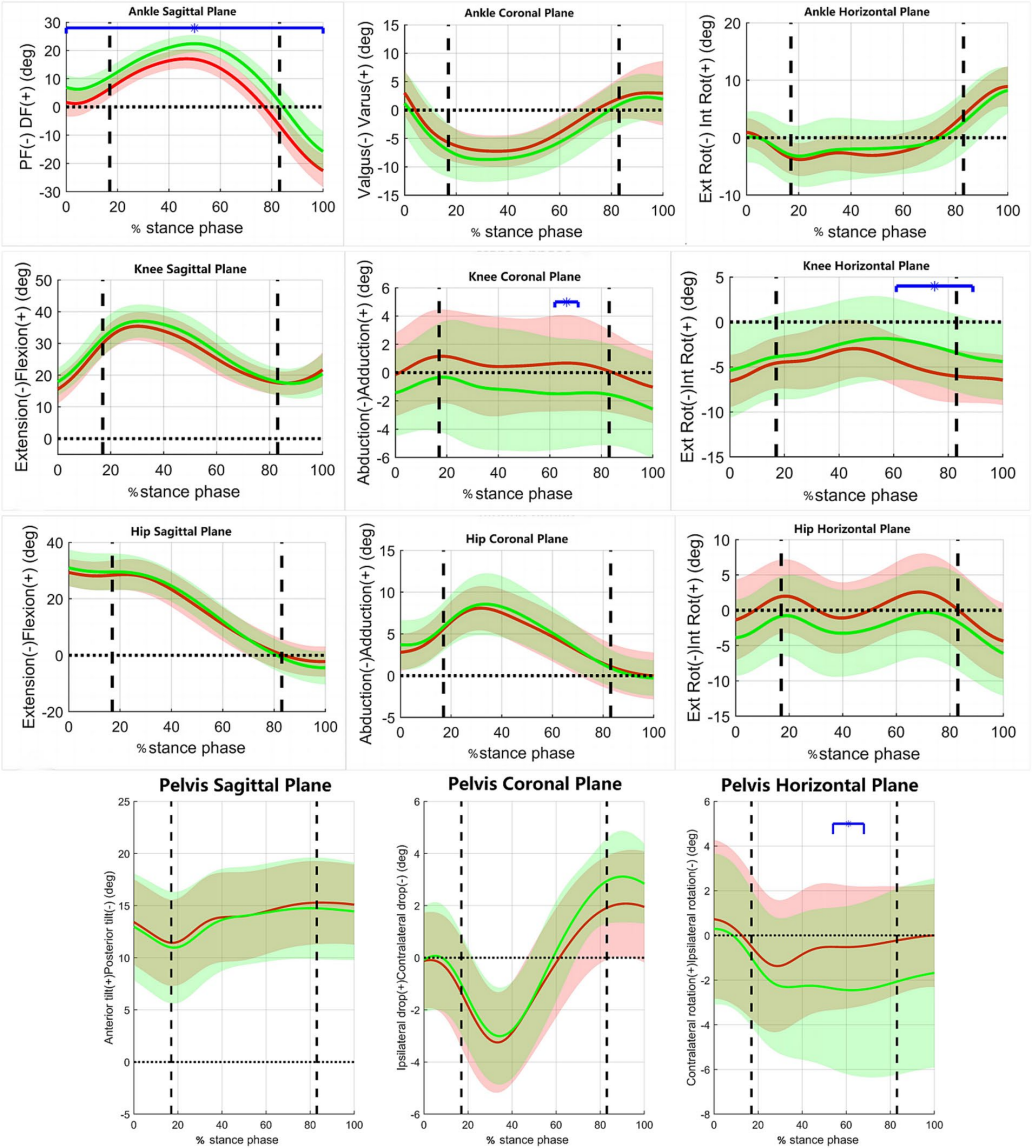
The variations of joint motion angles during jogging in the two groups of subjects. *x*-axis, the percentage of the stance phase of gait; *y*-axis, Joint angles (°); Red Line, RADF group; Green Line, un-RADF group; Blue horizontal line, significant effect; DF-dorsiflexion, PF-plantarflexion; Ext Rot, External Rotation; Int Rot, Internal Rotation. First vertical dotted line, contralateral toe off; Second vertical dotted line, contralateral heel off.

**Figure 4:**
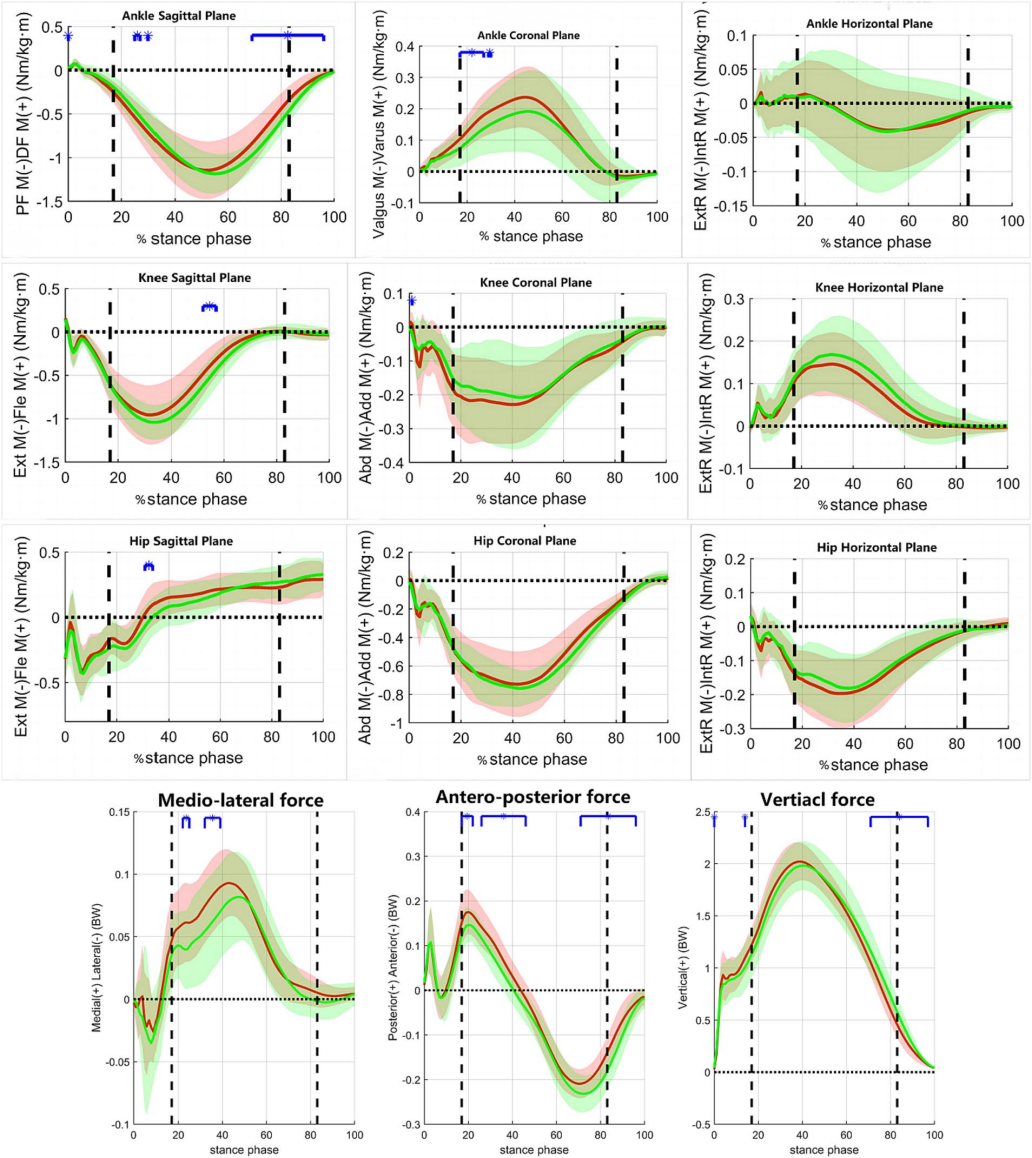
The variations of moments and ground-reaction force during walking in the two groups of subjects. *x*-axis, the percentage of the stance phase of gait; *y*-axis, the moment of force/Ground reaction force; Red Line, RADF group; Green Line, un-RADF group; Blue horizontal line, significant effect; BW, body weight; BW * BH, body weight multiplied by body height; Ext M, Extension moment; Fle M, Flexion moment; ExtR M, External Rotation moment; IntR M, Internal Rotation moment. First vertical dotted line, contralateral toe off; Second vertical dotted line, contralateral heel off.

Figure 3 shows the variations in the joint motion angles of the pelvis and lower limbs in the coronal, sagittal, and horizontal planes during the stance phase of the jogging process in both groups of subjects. Figure 4 shows the variations of moments in the coronal, sagittal, and horizontal planes of each joint of the lower extremity during jogging in the two groups of subjects. Table 3 shows the results of comparing the lower limb biomechanical parameters corresponding to the moment of peak ankle dorsiflexion angle during the stance phase, and the parameters that were significantly smaller in the RADF group than in the un-RADF group were: peak ankle dorsiflexion angle (RADF group: 17.22 ± 3.43°, un-RADF group: 22.79 ± 2.98°, *p* < 0.01); the anterior ground reaction force corresponding to this peak moment (RADF group: 0.02 ± 0.03 BW, un-RADF group: 0.06 ± 0.04 BW, *p* < 0.01), and the angle of pelvic ipsilateral rotation (RADF group: 0.65 ± 2.89°, un-RADF group: 2.56 ± 3.77°, *p* < 0.05). No significant differences were found between other biomechanical parameters of the lower extremities and the pelvis.

**Table 3:** Biomechanical parameters during the stance phase during jogging.

Variables	un-RADF group (SD)	RADF group (SD)	*t*-value	*p*-value	Mean difference (95% CI)
Ankle					
Ankle dorsiflexion (°)	22.79 (2.98)	17.22 (3.43)	6.01	<0.001*	−5.57 (−7.43 to −3.71)
Ankle eversion (°)	7.74 (3.85)	6.69 (2.89)	1.05	0.30	1.04 (−0.97 to 3.06)
Ankle abduction (°)	2.02 (5.15)	2.92 (3.24)	−0.71	0.48	−0.90 (−3.49 to 1.69)
Ankle moment					
Ankle plantarflexion moment (BW * BH)	1.16 (0.20)	1.13 (0.33)	0.39	0.70	0.03 (−0.13 to 0.19)
Ankle varus moment (BW * BH)	0.19 (0.14)	0.23 (0.10)	−1.30	0.20	0.04 (−0.02 to 0.11)
Ankle adduction moment (BW * BH)	0.04 (0.09)	0.04 (0.06)	−0.17	0.86	−0.004 (−0.05 to 0.04)
Knee					
Knee flexion (°)	32.51 (5.40)	31.56 (4.87)	0.65	0.52	−0.94 (−3.86 to 1.97)
Knee abduction (°)	1.42 (4.38)	−0.4 (3.38)	1.68	0.10	1.82 (−0.36 to 4.00)
Knee external rotation (°)	2.2 (4.77)	2.94 (3.08)	−0.62	0.54	−0.74 (−3.15 to 1.67)
Knee moment					
Knee flexion moment (BW * BH)	0.7 (0.20)	0.66 (0.27)	0.54	0.59	0.04 (−0.10 to 0.18)
Knee abduction moment (BW * BH)	0.19 (0.15)	0.23 (0.11)	−1.00	0.33	−0.04 (−0.11 to 0.04)
Knee internal rotation moment (BW * BH)	0.12 (0.09)	0.1 (0.07)	0.83	0.41	−0.02 (−0.06 to 0.03)
Hip					
Hip flexion (°)	18.66 (5.36)	19.23 (5.77)	−0.36	0.72	0.57 (−2.64 to 3.78)
Hip adduction (°)	6.86 (3.19)	6.78 (2.73)	0.09	0.93	−0.08 (−1.75 to 1.60)
Hip external rotation (°)	2.81 (6.19)	0.65 (4.93)	1.38	0.17	2.15 (−0.98 to 5.29)
Hip moment					
Hip flexion moment (BW * BH)	0.14 (0.13)	0.16 (0.12)	−0.60	0.55	0.02 (−0.05 to 0.09)
Hip abduction moment (BW * BH)	0.74 (0.13)	0.73 (0.22)	0.20	0.84	0.01 (−0.10 to 0.12)
Hip external rotation moment (BW * BH)	0.14 (0.09)	0.17 (0.09)	−1.26	0.21	−0.03 (−0.09 to 0.02)
Pelvis					
Anterior pelvic tilt (°)	14 (4.67)	13.99 (4.31)	0.01	0.99	−0.01 (−2.56 to 2.54)
Pelvic ipsilateral tilt (°)	1.36 (1.89)	2.15 (1.88)	−1.48	0.14	−0.80 (−1.87 to 0.28)
Pelvis ipsilateral rotation (°)	2.56 (3.77)	0.65 (2.89)	2.04	0.047*	1.91 (0.03 to 3.78)
GRF					
Medial (BW)	0.09 (0.03)	0.09 (0.03)	−1.06	0.30	0.01 (−0.01 to 0.02)
Anterior (BW)	0.06 (0.04)	0.02 (0.03)	3.84	<0.001*	0.04 (0.02 to 0.06)
Vertical (BW)	1.86 (0.27)	1.92 (0.17)	−1.04	0.30	0.06 (−0.06 to 0.19)
Jogging velocity					
V (m/s)	2.39 (0.15)	2.30 (0.17)	1.80	0.08	−0.08 (−0.18 to 0.01)

SD, standard deviation; CI, Confidence Interval; *, significant effect; BW, body weight; BW * BH, body weight multiplied by body height; GRF, ground-reaction force; Medial, medial ground reaction force; Posterior, Posterior ground reaction force; Vertical, Vertical ground reaction force.

## 4. Discussion

The objective of this research was to investigate the biomechanical characteristics of the lower extremity in individuals with limited ankle dorsiflexion during walking and jogging. Based on the peak ankle dorsiflexion angle during the stance phase measured in the walk test, the subjects were grouped and the differences in pelvic kinematics and lower extremity biomechanics during walking and jogging were investigated in individuals with different peak ankle dorsiflexion angles during the stance phase of gait in the walk and jogging tests. The results showed that during walking, the angles of the pelvis, hip, knee, and ankle joints were significantly different and the dynamics of the foot and ground reaction forces in the RADF group compared with that in the un-RADF group. During jogging, the pelvis and foot angles were significantly reduced in the RADF group.

The results showed that there was a significant difference in pelvis kinematics during walking between the RADF group and the un-RADF group in the walking test. Specifically, the angle of pelvic tilt to the ipsilateral side was significantly smaller in the RADF group than in the un-RADF group. This result suggests that important motor changes in the pelvis can exist in individuals with reduced ankle mobility. In gait, the pelvis rotates in all three planes, helping to decrease the movement of the center of mass in the vertical and horizontal direction thus being energetically economical (29). The pelvic tilt is one of the determinants of the mediolateral displacement of the center of mass (COM) and also helps to reduce the vertical displacement of the center of gravity (30). Therefore, the reduction in the angle of ipsilateral tilt of the pelvis in the group with limited ankle dorsiflexion affects the change in the center of gravity in gait, which in turn has an impact on walking. Previous literature has reported that the horizontal plane motion of the pelvis occurs less during walking in those with limited ankle dorsiflexion compared to those without (15), whereas the literature has rarely addressed the frontal plane motion of the pelvis, so this study extends the study of the effect of limited ankle dorsiflexion mobility on the motion of the frontal plane of the pelvis, that is the angle of the pelvis tilted to the ipsilateral side during walking was significantly less in the group with limited ankle dorsiflexion than in the non-limited group. During jogging, the angle of pelvic rotation to the ipsilateral side was significantly smaller in the group with restricted ankle dorsiflexion than in the unrestricted group (*p* < 0.05). The results suggest that individuals with smaller ankle dorsiflexion angles will have less movement in the horizontal plane of the pelvis during exercise, and a previous study (31) has shown that the smaller the pelvic rotation relative to the supporting foot during the support phase of gait, the greater the torsional stress on the lower extremity, which correlates more with lower extremity injury (32).

The RADF group had a significantly lower hip extension angle in the walking test. It was indicated that limitation of ankle dorsiflexion was significantly associated with limitation of hip extension during walking. Peak ankle dorsiflexion occurs at the moment of heel lift at the end of the stance phase of gait when the hip is in extension (33). Ankle push-off contributes to leg swing and propels the body over the supporting lateral limb (24), while a decrease in peak ankle dorsiflexion may decrease ankle stirrup strength and hip extension. Meanwhile, hip extension more appropriately loads the ankle in dorsiflexion, creating better muscular and mechanical energy, which is essential for stance-to-swing transition and thus forward propulsion (34). Therefore, the results of this study suggest that a reduction in peak ankle dorsiflexion affects the movement of the sagittal plane of the hip joint, which in turn adversely affects the transition from the stance to the swing phase in gait.

Differences in knee motion during walking were observed between the two groups of subjects, with the RADF group having a significantly greater angle of external knee rotation. The external rotation of the knee that occurs at the end of the support phase can be explained according to the “screw-home mechanism” (35), where the final extension of the knee during the gait cycle is normally accompanied by the external rotation of the tibia relative to the femur. In contrast, the RADF group showed greater external knee rotation at the moment of peak ankle dorsiflexion. When the knee joint is extended, the anterior cruciate ligament (ACL) gets tangled and tightened if the tibia is rotated externally with respect to the femur (screw-home movement) (36), which may increase the risk of ACL injury. This is because the ACL not only prevents knee hyperextension but also stabilizes the knee against tibial rotation (37). Many researchers have reported that knee rotation is significantly associated with ACL injury (38–41) and that external knee rotation combined with knee abduction may cause the ACL to impinge on the femoral condyle, which in turn increases the load on the ACL (42). Therefore, greater external knee rotation angles in individuals with limited ankle dorsiflexion may increase the risk of a knee injury. However, no changes in knee biomechanical parameters other than knee external rotation angle were found in this study, which is not consistent with the hypothesis of this study and the results in the literature (15, 43) and may be related to the different grouping methods and inter-subject differences.

In the present study, during the walking test, the RADF group had a smaller ankle dorsiflexion moment. Meanwhile, the RADF group also had smaller anterior ground reaction forces in both walking and jogging test. In gait, the body is propelled forward mainly through plantar flexion of the stirrups off the ground to generate thrust (32). In contrast, the plantarflexion push-off moment of the ankle joint is generated by the triceps calf muscle (biceps, medial and lateral gastrocnemius) and other external foot muscle-tendon units. And the peak ankle push-off force is partially derived from the release of elastic energy stored in the Achilles tendon during ankle dorsiflexion (44). The results of the study showed that a restricted ankle dorsiflexion angle reduces the ankle plantarflexion moment, which suggests that individuals with restricted ankle dorsiflexion have less ability to swing their lower limbs forward during walking. Also, this may account for the less forward ground reaction force in the RADF group during walking versus jogging. From the results, it was observed that the anterior ground reaction force of walking was greater than that of jogging, which may be caused by changes in gait parameters due to changes in movement patterns during the transition from walking to running, such as the duration of the stance phase and the change in stride frequency, as well as the choice of walking versus jogging speed that equally affects the magnitude of the ground reaction force, which is consistent with the results of previous studies in the literature (45). Since the medial-lateral forces have particularly high coefficients of variation (46–48), they are the least reliable among the ground reaction forces and therefore are not analyzed in this study for the time being.

## 5. Strengths and limitations

In this study, a three-dimensional motion capture system is proposed to determine whether subjects have sufficient ankle dorsiflexion angle to complete functional movements such as walking and jogging. This study also systematically analyzed the biomechanical effects of different ankle dorsiflexion angles on hip, knee, ankle and pelvis during walking and jogging.

The present study has several limitations. Based on the maximum dorsiflexion angle in walking test, this study proposed a novel method of diagnosing functional limited ankle dorsiflexion by maximum ankle dorsiflexion during stance phase of walking. However, this method was not further compared with other methods such as the weight-bearing lunge test, which may affect the validity of this method. This study focused on and discussed lower extremity biomechanics and pelvic motion during walking versus jogging in individuals with ankle dorsiflexion restrictions, but did not further compare the differences between walking and jogging in individuals with ankle dorsiflexion restrictions. Jogging requires a greater ankle dorsiflexion angle to propel the body forward, but the transition from walking to running shortens the duration of the support period of gait (38), which can affect the biomechanics of the lower extremity during the gait cycle and needs to be continued to be explored in future studies. In addition, lower extremity muscle activity or muscle strength was not assessed as a variable in this study and needs to be further advanced in future studies. Finally, due to the lack of upper limb model construction, the COM could not be determined, and the changes of the COM in individuals with different ankle dorsiflexion angles during walking and jogging should be further studied.

## 6. Conclusion

The present study demonstrated that during walking, individuals with Smaller ankle dorsiflexion peaks in gait result in reduced pelvic frontal plane motion; reduced hip posterior extension at the moment of peak ankle dorsiflexion, increased knee external rotation angle, and reduced ankle plantarflexion moment and anterior ground reaction force. During jogging, ipsilateral pelvic rotation and anterior ground reaction forces were reduced in those with limited ankle dorsiflexion. Thus, limited ankle dorsiflexion alters the movement pattern of the lower extremity during walking and jogging, diminishing the body’s ability to propel forward, which may lead to higher injury risks.

## Data availability statement

The original contributions presented in the study are included in the article/supplementary material, further inquiries can be directed to the corresponding authors.

## Ethics statement

The studies involving humans were approved by the Ethics Committee of Peking University Third Hospital. The studies were conducted in accordance with the local legislation and institutional requirements. The participants provided their written informed consent to participate in this study.

## Author contributions

YR: Writing – review & editing, Conceptualization, Methodology, Data curation, Formal analysis, Investigation, Project administration, Software, Supervision, Validation. NY: Writing – original draft, Data curation, Formal analysis, Investigation, Validation. TG: Writing – review & editing, Data curation, Formal analysis, Investigation, Validation. SZ: Validation, Writing – review & editing, Data curation, Formal analysis, Investigation. HS: Writing – review & editing, Data curation, Formal analysis, Investigation, Validation. YL: Writing – review & editing, Data curation, Formal analysis, Investigation, Validation. SR: Writing – review & editing, Conceptualization, Data curation, Formal analysis, Investigation, Methodology, Project administration, Software, Supervision, Validation. HH: Writing – review & editing, Conceptualization, Data curation, Formal analysis, Investigation, Methodology, Project administration, Software, Supervision, Validation.
